# Clinical implications and optimal extent of lymphadenectomy for intrahepatic cholangiocarcinoma: A multicenter analysis of the therapeutic index

**DOI:** 10.1002/ags3.12642

**Published:** 2022-11-27

**Authors:** Yuzo Umeda, Kosei Takagi, Tatsuo Matsuda, Tomokazu Fuji, Toru Kojima, Daisuke Satoh, Masayoshi Hioki, Yoshikatsu Endo, Masaru Inagaki, Masahiro Oishi, Takahito Yagi, Toshiyoshi Fujiwara

**Affiliations:** ^1^ Department of Gastroenterological Surgery Okayama University Graduate School of Medicine, Dentistry and Pharmaceutical Sciences Okayama Japan; ^2^ Department of Surgery Tenwakai Matsuda Hospital Kurashiki Japan; ^3^ Department of Surgery Okayama Saiseikai General Hospital Okayama Japan; ^4^ Department of Surgery Hiroshima Citizens Hospital Hiroshima Japan; ^5^ Department of Surgery Fukuyama City Hospital Hiroshima Japan; ^6^ Department of Surgery Himeji Red Cross Hospital Himeji Japan; ^7^ Department of Surgery National Hospital Organization Fukuyama Medical Center Fukuyama Japan; ^8^ Department of Surgery Tottori Municipal Hospital Tottori Japan

**Keywords:** intrahepatic cholangiocarcinoma, lymphadenectomy, multicenter study, retrospective study

## Abstract

**Aims:**

Lymph node metastases (LNM) are associated with lethal prognosis in intrahepatic cholangiocarcinoma (ICC). Lymphadenectomy is crucial for accurate staging and hopes of possible oncological treatment. However, the therapeutic implications and optimal extent of lymphadenectomy remain contentious.

**Methods:**

To clarify the prognostic value and optimal extent of lymphadenectomy, the therapeutic index (TI) for each lymph node was analyzed for 279 cases that had undergone lymphadenectomy in a multi‐institutional database. Tumor localization was divided into hilar lesions (n = 130), right peripheral lesions (n = 60), and left peripheral lesions (n = 89). In addition, the lymph node station was classified as Level 1 (LV1: hepatoduodenal ligament node), Level 2 (LV2: postpancreatic or common hepatic artery nodes), or Level 3 (LV3: gastrocardiac, left gastric artery, or celiac artery nodes).

**Results:**

Lymph node metastases were confirmed in 109 patients (39%). Five‐y survival rates were 45.3% for N0 disease, 27.1% for LV1‐LNM, 22.9% for LV2‐LNM, and 7.3% for LV3‐LNM (*P* < 0.001). LV3‐LNM were the most frequent and earliest recurrence outcome, including multisite recurrence, followed by LV2, LV1, and N0 disease. The 5‐year TI (5year‐TI) for lymphadenectomy was 7.2 for LV1, 5.5 for LV2, and 1.9 for LV3. Regarding tumor location, hilar lesions showed 5‐year TI >5.0 in LV1 and LV2, whereas bilateral peripheral lesions showed 5‐year TI > 5.0 in LV1.

**Conclusion:**

The implications and extent of lymphadenectomy for ICC appear to rely on tumor location. In the peripheral type, the benefit of lymphadenectomy would be limited and dissection beyond LV1 should be avoided, while in the hilar type, lymphadenectomy up to LV2 could be recommended.

## Graphical abstract

Lymph node metastases are lethal prognostic factors in intrahepatic cholangiocarcinoma (ICC). Lymphadenectomy is crucial for accurate staging and hoped‐for as possible oncological treatment in ICC. A prognostic value of lymphadenectomy could be proven by analysis of the therapeutic index, especially in the hilar type. Lymphadenectomy should be a minimal requirement for the multimodal treatment of ICC. The implication and extent of lymphadenectomy for ICC should rely on tumor location.

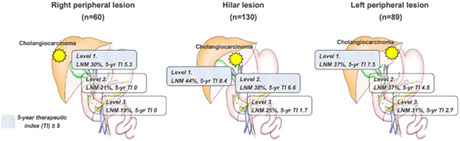

## 1 INTRODUCTION

Intrahepatic cholangiocarcinoma (ICC) is one of the primary hepatocellular carcinomas, second only to hepatocellular carcinoma in its incidence. ICC arises from the epithelial cells of either small intrahepatic ducts or large intrahepatic ducts near the bifurcation of intrahepatic ducts.^1^ In both clinical situations, the pathological findings are usually classified as adenocarcinoma, although mixed hepatocellular cholangiocarcinoma also occurs, particularly in chronic liver disease. Despite its rarity, ICC is difficult to detect and treat and tends to be advanced at diagnosis.

Surgical resection is the only established option for treating ICCs, offering the best chance of cure.^2^ However, because many patients with ICC have large, locally advanced tumors that require technically complex and challenging surgery, only about 20%–40% of patients with operable disease undergo surgical resection. In addition, the incidence of lymph node metastases (LNM) reportedly ranges from 17% to 62% and reflects a poor prognosis.^3^, ^4^, ^5^, ^6^ Despite LNM being a significant predictor of poor prognosis, the evidence of therapeutic benefit from lymph node dissection is insufficient and no consensus has been reached regarding whether lymphadenectomy should be routinely performed.

In terms of clinical implications, routine lymphadenectomy has been considered paramount for accurate staging and determination of prognosis and for guiding the postoperative treatment strategy.^2^, ^7^ Moreover, recent clinical studies have consistently shown the oncological efficacy of lymphadenectomy for ICC and have thus tended to support this approach.^8^, ^9^ However, clear evidence is lacking regarding the extent of lymphadenectomy required for accurate staging and the prognostic value of lymphadenectomy for each lymph node station. Moreover, ICCs invading the hilar structures tend to be associated with a higher incidence of LNM than peripherally located tumors.^10^, ^11^, ^12^ Basing the extent of routine lymphadenectomy for accurate staging and treatment on tumor localization may thus be preferable.

The present study therefore aimed to investigate the survival benefit of lymphadenectomy among patients undergoing resection for ICC using a therapeutic index. We also evaluated the optimal extent of lymph node dissection for accurate staging and treatment by assessing tumor activity based on tumor localization.

## 2 METHODS

### Study subjects

This multicenter, retrospective study initially included 416 patients who underwent curative radical resection for ICC between January 2000 and December 2016. The clinical database was collected from 15 hospitals of the Okayama study group of HBP surgery with an aggressive attitude toward lymphadenectomy for ICC, including 11 hospitals qualified as board‐certified training institutions for the Hepatobiliary and Pancreatic Surgery Program in Japan.^13^ Most patients were therefore collected from institutions with sufficient surgical cases, leading to reliable surgical techniques and outcomes. Moreover, we held the consensus meetings to determine the details of the operative techniques and extent of lymphadenectomy by discussing several operative videos to eliminate differences among institutions as possible. Consequently, data from 279 patients who underwent curative liver resection with systematic lymphadenectomy were analyzed, while the other 137 patients who did not undergo lymphadenectomy were excluded. The definition of each pathologic factor was established based on the General Rules for the Clinical and Pathological Study of Primary Liver Cancer.^14^

Concerning localization, all ICCs were classified as hilar or peripheral based on the anatomical origin of the tumor. The anatomical location of the tumor was judged from preoperative imaging such as computed tomography or magnetic resonance imaging. Primary tumors with a large proportion of the tumor in contact with the hepatic hilum (between the right side of the umbilical portion of the left portal vein and the left side of the origin of the right posterior portal vein) were defined as hilar‐type ICC, whereas other tumors without these contacts were defined as peripheral‐type ICC. Moreover, in cases of multiple tumors localization was classified according to the locus of the largest tumor.

### Assessment of lymph node metastasis and therapeutic index of lymphadenectomy

Lymph node metastases were assessed according to “Classification of Biliary Tract Cancers established by the Japanese Society of Hepato‐Biliary‐Pancreatic Surgery: Third Edition.”^15^ Furthermore, lymph nodes were then classified into three levels from the hepatic hilum to the distal side of the liver: Level 1, hepatoduodenal ligament node; Level 2, postpancreatic head or common hepatic artery nodes; and Level 3, gastrocardiac, left gastric artery, or celiac artery nodes. Moreover, the extent of LNM was judged from the most distal node from the hepatic hilum, with the distribution of LNM classified into a more significant number of levels. To evaluate the therapeutic value of lymphadenectomy at any nodal station, we determined the frequency of metastasis at that nodal station and calculated the cumulative 2‐ and 5‐year overall survival rates of patients with metastasis at that nodal station, irrespective of the presence/absence of metastasis at other nodal stations. Next, we calculated the frequency of LNM, with the number of dissected cases as the denominator and the number of positive cases of LN metastasis as the numerator. Finally, the frequency of LNM was multiplied by the 2‐ and 5‐year survival rates of patients with LNM to calculate a therapeutic index (TI) of benefit from lymphadenectomy at each station.^16^ Based on these results, we recommended lymphadenectomy when the 5‐year TI was significant for indices exceeding 5.0.

### 2.2 Statistical analysis

Clinical variables were compared using the Mann–Whitney *U*‐test for continuous data and Pearson's correlation coefficient for categorical data. Continuous variables are presented as the median and interquartile range (IQR). Values of *P* < 0.05 were considered significant. Survival curves were estimated using Kaplan–Meier methods for survival analysis, and differences in survival were evaluated with the log‐rank test. In addition, we identified risk factors for LNM by logistic regression analysis and overall survival by the Cox proportional hazard model. Clinical variables showing values of *P* < 0.10 in univariate analyses were entered into multivariate analysis for these analyses. All statistical analyses were performed using JMP v 14 (SAS Institute, Cary, NC, USA).

## 3 RESULTS

Overall patient background characteristics and differences between tumor locations are summarized in Table 1. The median duration of follow‐up was 26.7 months interquartile range [IQR] 12.2–51.0 months). Median age at surgery was 70 years (IQR 63–76 years). The predominant tumor morphology was the mass‐forming type (MF; n = 198), followed by the MF and periductal infiltrating type (PI; n = 42), the PI type (n = 30), and the intraductal growth type (n = 9). Regarding tumor location, all ICCs were distributed to the right peripheral lesion (n = 60), hilar lesion (n = 130), and left peripheral lesion (n = 89). Compared with peripheral lesions, hilar lesions required bile duct resection and vascular reconstruction, leading to a longer operation time and larger blood loss as surgical outcomes. The extent of lymph node dissection varied between centers. Of the 15 participating institutions, 12 institutions had performed lymphadenectomy beyond Level 1 in 80% of cases. The other three institutions also performed the same extended dissection in 40%–50% of cases. (Figure S1). Of the 279 patients who underwent lymphadenectomy, 109 patients (39%) showed histologically confirmed LNM. According to tumor location, the highest rates of both LNM and LNM beyond Level 1 were seen in the hilar lesion, followed by the left peripheral lesion and right peripheral lesion. The extent of LNM comprised Level 1‐LNM (n = 31, 11%), Level 2‐LNM (n = 44, 16%), and Level 3‐LNM (n = 34, 12%). In the distribution of LNM, some overlap in cases was evident. All patients classified with Level 2‐LNM were identified as showing LNM in Level 1. On the other hand, among patients classified into Level 3‐LNM, two patients showed no evidence of positive nodes in Level 2, and one patient showed positive nodes in neither Level 1 nor Level 2 (Figure S2). Considering postoperative morbidity after lymphadenectomy, rates of severe complications (defined as Clavien–Dindo [CD] >grade II) were comparable between lymphadenectomies for Levels 1, 2, and 3. The rates of CD >grade II were 17% in Level 1, 19% in Level 2, and 18% in Level 3, respectively (data not shown, *P* = 0.168). However, CD grade II, represented by gastric stasis, was prominent in lymphadenectomy for Level 3, occurring in 19 cases for Level 3, only one case for Level 2, and no case for Level 1.

**TABLE 1:** Overall patient characteristics and differences between tumor locations

Variables	Overall (n = 279)	Tumor location			*P*‐value^a^
		Right peripheral lesion	Hilar lesion	Left peripheral lesion	
		(n = 60)	(n = 130)	(n = 89)	
Preoperative factors					
Male, n (%)	159 (57%)	33 (58%)	76 (58%)	50 (56%)	0.889
Age (years), median (IQR)	70 (63–76)	69 (59–76)	70 (65–77)	68 (63–76)	0.314
Tumor factors					
Morphology, n (%)					
MF	198 (71%)	51 (85%)	80 (62%)	67 (75%)	0.002
PI	30 (11%)	4 (6%)	18 (14%)	8 (9%)	
MF + PI	42 (15%)	4 (6%)	30 (23%)	8 (9%)	
IG	9 (3%)	1 (2%)	2 (2%)	6 (7%)	
Tumor size (cm), median (IQR)	4.5 (3–7)	6 (3.9–8)	4 (2.8–6.8)	4.5 (2.8–6.8)	0.004
Multi‐nodular, n (%)	64 (23%)	23 (38%)	24 (19%)	17 (19%)	0.006
LNM‐radiological image, n (%)	101 (36%)	10 (17%)	53 (41%)	38 (43%)	0.002
CEA (ng/ml), median (IQR)	2.9 (1.8–5.9)	2.7 (1.8–7.8)	3.1 (2–6.5)	2.7 (1.7–4.4)	0.151
CA19–9 (U/ml), median (IQR)	51.5 (16–314)	45.4 (15.6–260)	65.6 (19.4–1019)	33.7 (13.5–151)	0.051
Operative factors					
Major hepatectomy, n (%)	239 (86%)	37 (62%)	124 (95%)	78 (88%)	<0.001
Bile duct resection, n (%)	109 (39%)	4 (6%)	96 (74%)	9 (10%)	<0.001
Vascular reconstruction^b^, n (%)	28 (10%)	2 (3%)	24 (18%)	2 (2%)	<0.001
Blood loss (ml), median (IQR)	810 (402–1377)	975 (520–1400)	920 (497–1712)	545 (296–1015)	<0.001
Operation time (min), median (IQR)	390 (300–485)	348 (300–428)	420 (360–519)	330 (250–425)	<0.001
Pathological factors					
Serosa invasion, n (%)	131 (47%)	24 (40%)	63 (48%)	44 (49%)	0.471
Microvascular invasion, n (%)	157 (56%)	31 (52%	76 (58%)	50 (56%)	0.680
LNM, n (%)	109 (39%)	18 (30%)	58 (45%)	33 (37%)	0.142
Extent of LNM, n (%)					
N0 disease	170 (61%)	42 (70%)	72 (55%)	56 (63%)	0.026
Level 1	31 (11%)	9 (15%)	14 (11%)	8 (9%)	
Level 2	44 (16%)	6 (10%)	29 (22%)	9 (10%)	
Level 3	34 (12%)	3 (5%)	15 (12%)	16 (18%)	
Differentiation, n (%)					
Well	66 (24%)	11 (18%)	35 (27%)	20 (22%)	0.218
Moderate/poor	195 (70%)	43 (72%)	91 (70%)	61 (69%)	
Unclassified	18 (6%)	6 (10%)	4 (3%)	8 (9%)	
Background liver, n (%)					
Normal	211 (76%)	44 (73%)	100 (77%)	67 (75%)	0.181
Hepatitis	44 (16%)	10 (17%)	21 (16%)	13 (15%)	
Fibrosis	11 (4%)	5 (8%)	1 (1%)	5 (5%)	
Unknown	13 (5%)	1 (2%)	8 (6%)	4 (4%)	

Abbreviations: BMI, body mass index; CA19‐9, carbohydrate antigen 19–9; CEA, carcinoembryonic antigen; IQR, interquartile range; LNM, lymph node metastasis; MF, mass‐forming; IG, intraductal growth; PI, periductal infiltrating; PNI, prognostic nutritional index.

^a^ Comparing between three groups.

^b^ Reconstruction for portal vein, hepatic artery, hepatic vein, and inferior vena cava.

**FIGURE 1:**
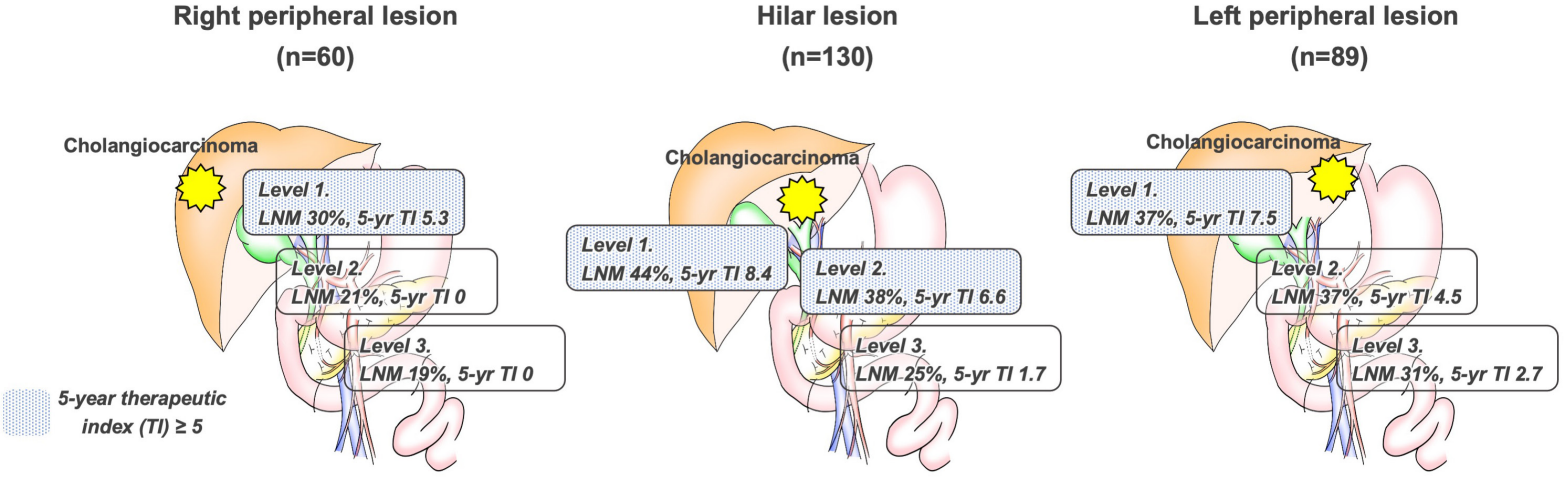
Overview of the 5‐year therapeutic index according to tumor location

In risk analysis for LNM by using patient background and tumor factor, logistic regression analysis identified “component of PI” (odds ratio [OR] 2.90, *P* = 0.002), “preoperative CA19‐9 level ≥ 118 U/ml” (OR 6.77, *P* < 0.001), “serosa invasion” (OR 1.81, *P* = 0.043), and “moderate or poor differentiation” (OR 4.51, *P* = 0.001) as independent risk factors for LNM (Table S1). An overview of LNM rates for each lymph node station and the therapeutic value of lymphadenectomy is provided in Table 2. Concerning TI for the overall cohort, 2‐/5‐y TI by lymphadenectomy were 12.8/7.2 for Level 1, 10.7/5.5 for Level 2, and 4.8/1.9 for Level 3. Subgroup analysis revealed that 5‐y TI varied between tumor locations; the 5‐y TI of lymphadenectomy in the hilar lesion was 4 for Level 1, 6.6 for Level 2, and 1.7 for Level 3. Moreover, the left peripheral lesion showed TIs of 7.5 for Level 1, 4.5 for Level 2, and 2.7 for Level 3. Particularly in the left peripheral lesion, incidences of metastasis in left gastric artery nodes and gastrocardiac nodes were 27.8% and 21.7%, respectively. Although the 2‐y TI for these nodes was approximately 10, the 5‐y TI was only 2.7. On the other hand, the right peripheral lesion contributed to 5‐y TI only for Level 1 (Figure 1).

**TABLE 2:** Therapeutic value of lymph node dissection

Zoning for Lymph node	Overall (n = 297)			Tumor location								
				Right peripheral lesion (n = 60)			Hilar lesion (n = 130)			Left peripheral lesion (n = 89)		
	Metastatic incidence	2‐/5‐year SR of node‐positive patients (%)	2‐/5‐year TI	Metastatic incidence	2‐/5‐year SR of node‐positive patients (%)	2‐/5‐year TI	Metastatic incidence	2‐/5‐year SR of node‐positive patients (%)	2‐/5‐year TI	Metastatic incidence	2‐/5‐year SR of node‐positive patients (%)	2‐/5‐year TI
Level 1												
Hilar and Hepatoduodenal ligament node (#12)	0.39 (108/279)	33.1/18.6	12.8/7.2	0.30 (18/60)	17.7/17.7	5.3/5.3	0.44 (57/130)	29.7/19.1	13.0/8.4	0.37 (33/89)	45.9/20.1	17.0/7.5
Level 2	0.35 (75/217)	30.9/15.8	10.7/5.5	0.21 (8/39)	25.0/0	5.1/0	0.38 (42/110)	26.1/17.4	9.9/6.6	0.37 (25/68)	41.1/12.4	15.1/4.5
Common hepatic artery node (#8)	0.29 (60/205)	30.5/13.4	8.9/3.9	0.17 (6/36)	33.3/33.3	5.6/5.6	0.34 (35/104)	19.4/11.7	6.5/3.9	0.29 (19/65)	45.8/13.1	13.4/3.8
Posterior pancreas head node (#13)	0.18 (18/101)	34.2/27.3	6.1/4.9	0.16 (3/19)	19.0/0	3.0/0	0.20 (11/54)	36.4/27.3	7.4/5.6	0.14 (4/28)	66.6/N/A	9.5/N/A
Level 3	0.27 (34/128)	18.1/7.3	4.8/1.9	0.19 (3/16)	0/0	0/0	0.25 (15/61)	6.7/6.7	1.7/1.7	0.31 (16/51)	33.9/8.5	10.6/2.7
Gastrocardiac node (#1, 3, and 5)	0.19 (18/93)	24.2/0	4.7/0	0.00 (0/6)	N/A	N/A	0.20 (8/41)	0/0	0/0	0.22 (10/46)	46.3/0	10.1/0
Left gastric artery and Celiac artery node (#7 and 9)	0.24 (19/80)	25.1/16.7	6.0/4.0	0.00 (0/6)	N/A	N/A	0.24 (9/38)	11.1/11.1	2.6/2.6	0.28 (10/36)	39.4 / 19.7	10.9/5.5
Para‐Aortic node (#16)	0.30 (9/30)	0/0	0/0	0.38 (3/8)	0/0	0/0	0.24 (4/17)	0/0	0/0	0.40 (2/5)	0/0	0/0

Abbreviations: LNM, lymph node metastasis; SR, survival rate; TI, therapeutic index.

**FIGURE 2:**
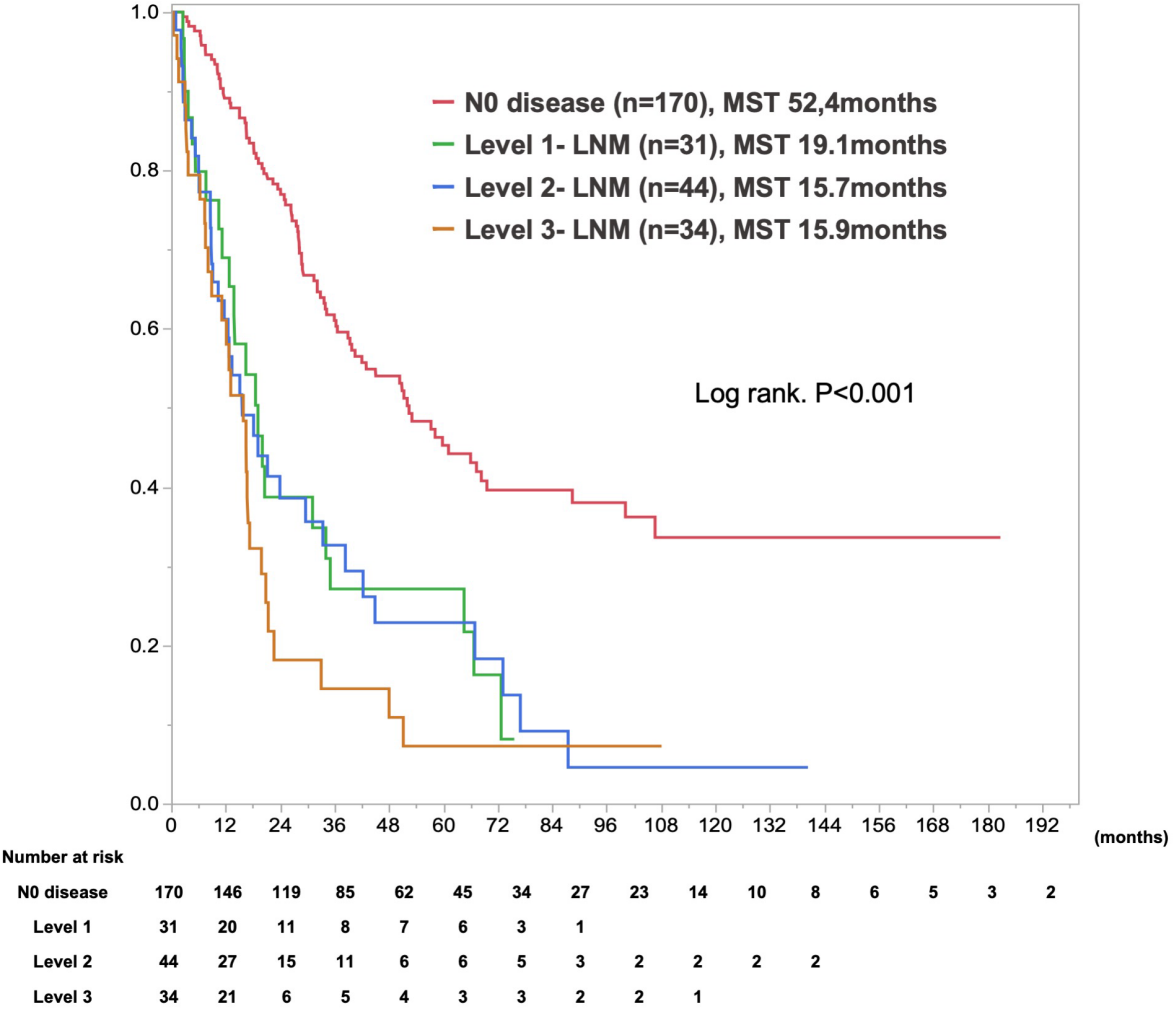
Kaplan–Meier curves for postoperative overall survival, stratified by level of lymph node metastasis

In survival analysis, median survival time (MST) and 1‐/5‐year overall survival rates after initial surgery were 52.4 months and 89.1%/45.3% for N0 disease, 19.1 months and 68.9%/27.1% for Level 1‐LNM, 15.7  months and 61.2%/22.9% for Level 2‐LNM, and 15.9 months, and 64.1%/7.3% for Level 3‐LNM (*P* < 0.0001; Figure 2). Level 3‐LNM showed the worst outcomes compared with the others. Conversely, the prognoses of Level 1‐LNM and Level 2 were comparable. In subgroup analysis stratified by tumor location, the prognoses of LNM from Level 1 to Level 3 were comparable for the right and left peripheral lesion (Figure S3).

**FIGURE 3:**
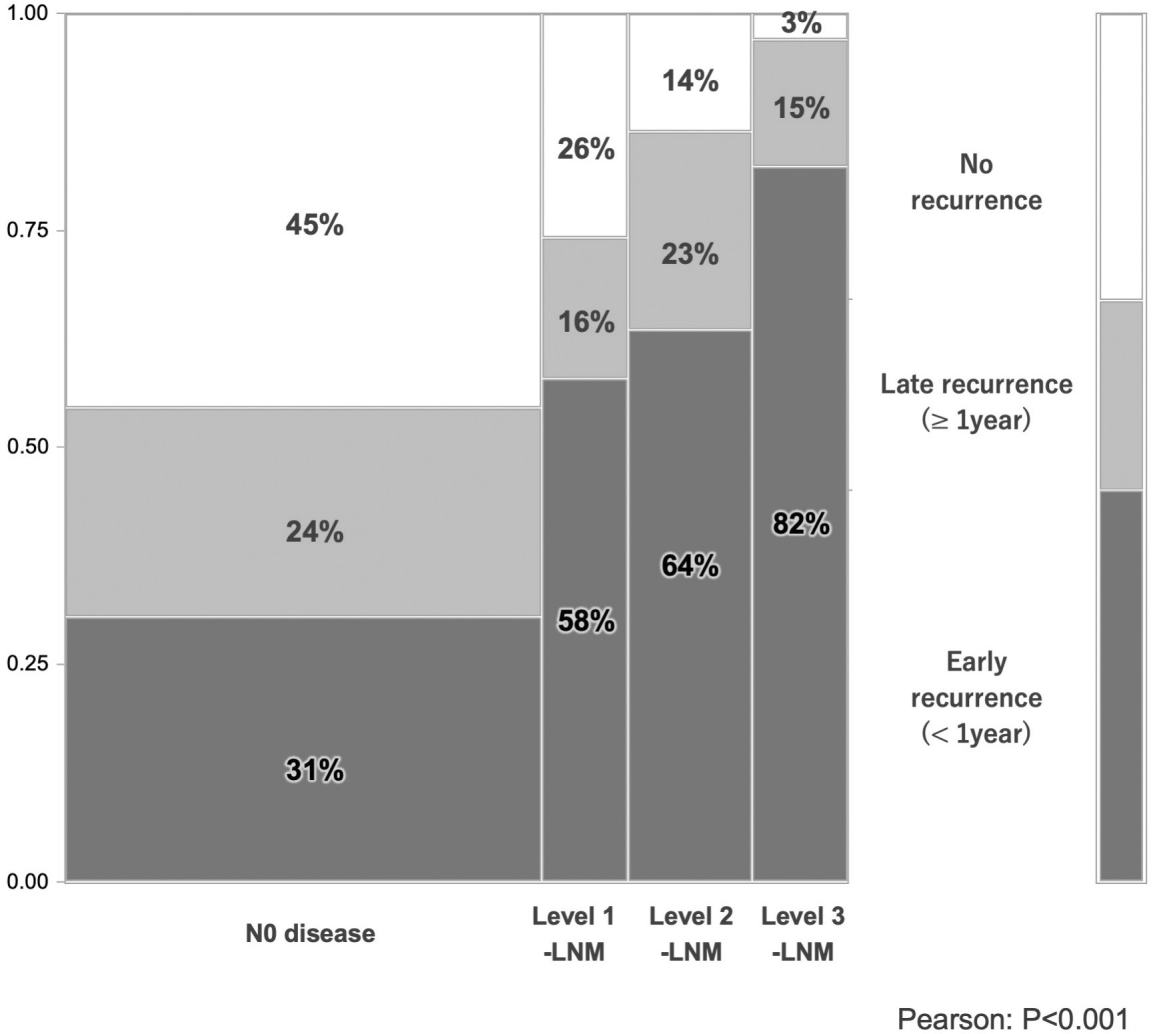
Proportion of recurrent timing in each level of lymph node metastasis

**FIGURE 4:**
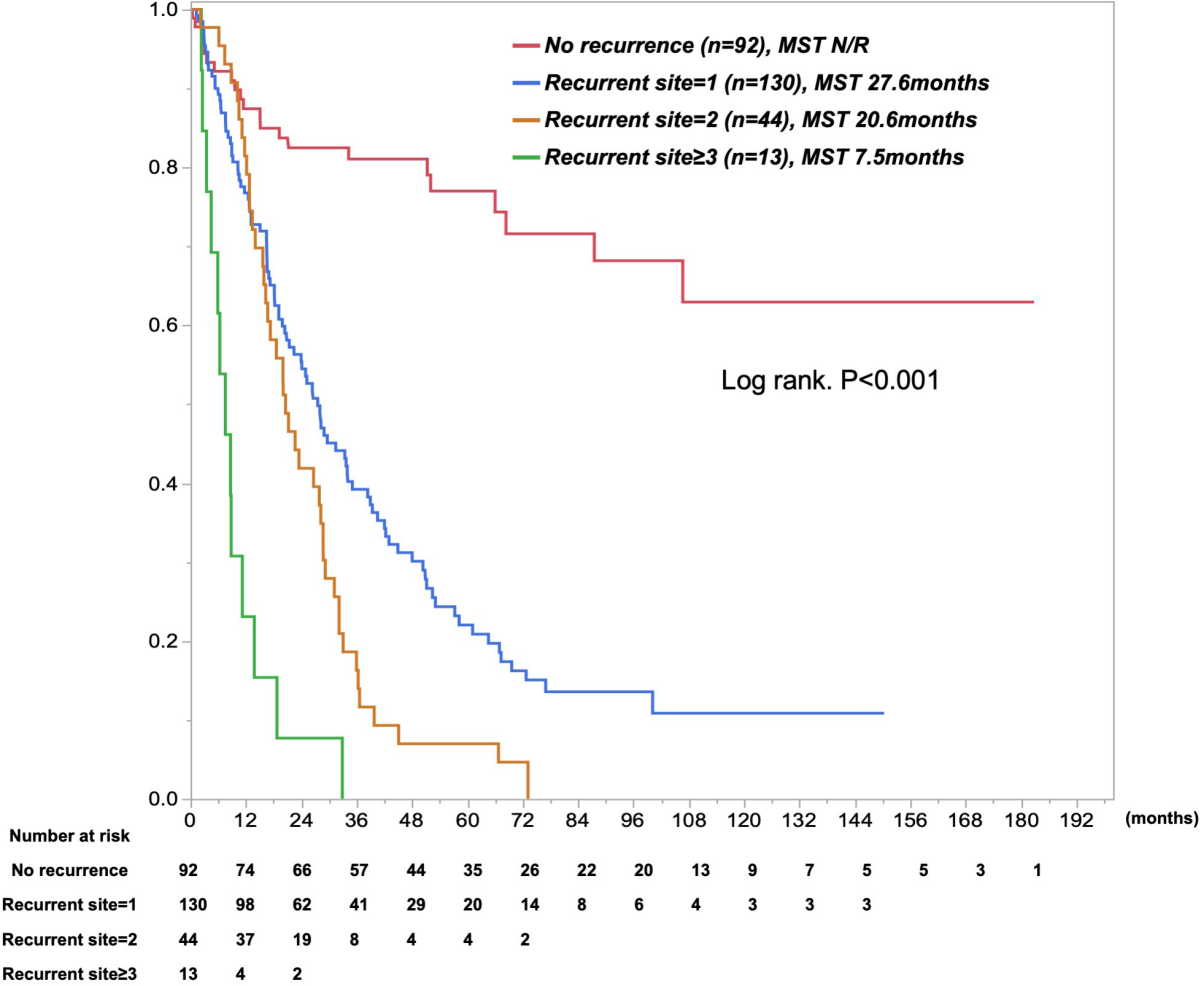
Kaplan–Meier curves for postoperative overall survival, stratified by number of recurrent sites

Concerning recurrent outcomes after surgery, N0 disease showed longer recurrence‐free survival (RFS) than did Level 1‐LNM, Level 2‐LNM, and Level 3‐LNM: median RFS was 29.8 months for N0 disease, 8.3 months for Level 1‐LNM, 9.2 months for Level 2, and 6 months for Level 3 (*P* < 0.001, Table 3). Furthermore, the highest early recurrence rate was seen for Level 3‐LNM, followed by Level 2‐LNM, Level 1‐LNM, and N0 disease (*P* < 0.001, Figure 3). The proportion of extrahepatic spread as recurrence increased with the extent of LNM (*P* = 0.083, Table 3). Most patients with Level 2‐ or Level 3‐LNM showed both early recurrence within 1 year after surgery and extrahepatic recurrence. Level 2‐ and Level 3‐LNM showed multisite recurrence, with an average of 1.3 recurrent sites in Level 3‐LNM, 1.2 recurrent sites in Level 2‐LNM, 1.2 recurrent sites in Level 1‐LNM, and 0.7 recurrent sites in N0 disease (*P* < 0.001, Table 3). Approximately 30% of recurrent patients with Level 2‐ or Level 3‐LNM showed 2–4 sites of recurrence, including remnant liver, lungs, peritoneum/pleura, and LNM. Reflecting these results, surgical resection rates for recurrent disease were 15% with N0 disease, 13% with Level 1‐LNM, 5% with Level 2‐LNM, and 3% with Level 3‐LNM. The proportion of best supportive care was increased by the extent of LNM. Survival curves for patients were categorized according to the number of sites of recurrence (Figure 4). In the Cox proportional hazard model, “Tumor size≥5 cm” (hazard ratio [HR] 1.48, *P* = 0.025), “Hilar lesion” (HR 1.55, *P* = 0.08), “Multinodular” (HR 1.46, *P* = 0.048), “CA19‐9 ≥ 118 U/ml” (HR 1.71, *P* = 0.004), and “Extent of LNM” (HR 1.82–2.89, *P* < 0.01) were identified as independent poor prognostic factors (Table 4). In particular, the prognostic impacts of the extent of Level 1‐, Level 2‐, and Level 3‐LNM were well categorized.

**TABLE 3:** Recurrence outcome by extent of lymph node metastasis

Variables	Extent of lymph node metastasis				*P*‐value^a^
	N0 disease	Level 1‐LNM	Level 2‐LNM	Level 3‐LNM	
	(n = 170)	(n = 31)	(n = 44)	(n = 34)	
Recurrence‐free survival (month), MST	29.8	8.3	9.2	6	<0.001
Timing of recurrence, n (%)					
No recurrence	77 (45%)	8 (26%)	6 (14%)	1 (3%)	<0.001
Early recurrence (<1 year)	52 (31%)	18 (58%)	28 (63%)	28 (82%)	
Late recurrence (≥1 year)	41 (24%)	5 (16%)	10 (23%)	5 (15%)	
Type of recurrence, n (%)					
Intrahepatic recurrence	30 (32%)	7 (30%)	10 (26%)	4(12%)	0.083
Intra‐ & extrahepatic recurrence	15 (16%)	7 (30%)	13 (34%)	12 (36%)	
Extrahepatic recurrence	48 (52%)	9 (39%)	15 (39%)	17 (52%)	
Site of recurrence, n (%)^b^					
Remnant liver	45 (27%)	14 (45%)	24 (55%)	18 (53%)	<0.001
Pleural/Peritoneum	25 (15%)	8 (26%)	11 (25%)	9 (26%)	0.155
Lymph node	15 (9%)	6 (19%)	11 (25%)	10 (29%)	0.002
Lung	22 (13%)	6 (19%)	5 (11%)	5 (15%)	0.763
Bone, etc	16 (9%)	4 (13%)	1 (2%)	3 (9%)	0.373
Number of recurrent site, mean	0.7	1.19	1.23	1.32	<0.001
Initial treatment for recurrence, n (%)					
Surgical resection	14 (15%)	3 (13%)	2 (5%)	1 (3%)	0.053
Radiation or RFA	7 (7%)	2 (9%)	5 (13%)	1 (3%)	
Chemotherapy	54 (58%)	11 (48%)	14 (37%)	14 (42%)	
BSC	15 (16%)	6 (26%)	15 (39%)	15 (45%)	
Unclassified	3 (3%)	1 (4%)	2 (5%)	2 (6%)	

Abbreviations: BSC, best supportive care; LNM, lymph node metastasis; MST, median survival time; RFA, radiofrequency ablation.

^a^ Comparing between four groups.

^b^ Counting all recurrence sites.

**TABLE 4:** Univariate and multivariate analysis of risk associated with overall survival after hepatectomy (n = 279)

Variables	Univariate analysis			Multivariate analysis		
	HR	95% CI	*P*‐value	HR	95% CI	*P*‐value
Background factor						
Gender						
Male	1.00 (reference)					
Female	0.94	0.69–1.27	0.701			
Age						
<60 years	1.00 (reference)					
≥60 years	1.26	0.79–2.01	0.328			
Tumor factor						
Tumor morphology						
MF	1.00 (reference)					
PI	1.29	0.81–2.05	0.286			
Tumor size						
<5 cm	1.00 (reference)			1.00 (reference)		
≥5 cm	1.37	1.00–1.89	0.046	1.48	1.05–2.10	0.025
Tumor location						
Peripheral	1.00 (reference)			1.00 (reference)		
Hilar	1.62	1.19–2.18	0.002	1.55	1.12–2.16	0.008
Multinodular						
No	1.00 (reference)			1.00 (reference)		
Yes	1.51	1.08–2.13	0.017	1.46	1.00–2.13	0.048
CA19‐9						
<118 U/ml	1.00 (reference)			1.00 (reference)		
≥118 U/ml	2.24	1.62–3.09	<0.001	1.71	1.18–2.47	0.004
Serosa invasion						
No	1.00 (reference)			1.00 (reference)		
Yes	1.75	1.30–2.37	<0.001	1.34	0.98–1.85	0.064
Vascular invasion						
No	1.00 (reference)			1.00 (reference)		
Yes	1.42	1.05–1.93	0.024	1.15	0.83–1.60	1.152
Extent of LNM						
N0	1.00 (reference)			1.00 (reference)		
Level 1	2.48	1.55–3.94	<0.001	1.82	1.11–3.01	0.018
Level 2	2.72	1.83–4.04	<0.001	1.90	1.23–2.96	0.004
Level 3	3.79	2.47–5.82	<0.001	2.89	1.81–4.64	<0.001
Differentiation						
Well	1.00 (reference)	1.05–1.64		1.00 (reference)	1.05–1.64	
Moderate/poor	1.69	1.15–2.49	0.008	1.22	0.80–1.86	0.354
Unclassified	1.35	0.66‐‐2.75	0.406	1.24	0.59–2.58	0.564

Abbreviations: BMI, body mass index; CA19‐9, carbohydrate antigen 19–9; CI, confidence interval; HR, hazard ratio; LNM, lymph node metastasis; MF, mass‐forming; PI, periductal infiltrating.

## 4 DISCUSSION

Routine lymphadenectomy for ICC has been controversial because of the lack of clinical evidence. Nevertheless, recent clinical studies have supported lymphadenectomy for oncological treatment and staging.^8^, ^9^ Thus, in current trends, the proportion of patients undergoing lymph node dissection for ICC has increased year by year, particularly in Western countries.^17^ However, specifics of the scope and methods of lymphadenectomy have yet to be defined. The American Joint Committee on Cancer (AJCC) recommends harvesting at least six lymph nodes to ensure accurate staging.^18^ International collaborative studies were concordant with this recommendation.^19^, ^20^ The National Comprehensive Cancer Network guidelines also recommend regional lymphadenectomy in the hilar region of the liver, while AJCC guidelines recommend a broader level, depending on tumor location.^18^, ^21^ According to the AJCC guidelines, lymphadenectomy should be performed beyond the hepato‐duodenal ligament, depending on the localization of the tumor. However, this proposal lacks sufficient evidence‐based data and is instead based on expert recommendations. The present study aimed to evaluate the efficacy and significance of lymph node dissection for ICC and to define the optimal extent of lymph node dissection according to tumor localization. With constraints such as the relative rarity of ICC and the commonly accepted surgical strategy of lymphadenectomy, establishing a randomized controlled study would be invaluable, but unrealistic. Some previous studies have reported the therapeutic benefit of lymphadenectomy for ICC.^8^, ^9^, ^22^ However, those studies were mainly retrospective studies using simulation analysis, propensity score matching, and inverse probability of treatment‐weighting approaches with propensity scores. The present study focused on TI as a promising index with proven implications in gastric cancer, where the efficacy of lymph node dissection is well established.^16^, ^23^ The significance of TI has been demonstrated more recently for various gastrointestinal cancers, including colorectal cancer, esophageal cancer, and pancreatic cancer.^24^, ^25^, ^26^, ^27^ Sahara et al^28^ reported on the significance of TI for ICC, and identified which patients would benefit from lymphadenectomy.

Contrasting with their report, we focused on tumor location and set the valuable index to 5 y. This was because ICC has different aspects of lymph node metastasis, depending on tumor location^11^, ^22^, ^29^, ^30^ and we wished to evaluate the efficacy of lymphadenectomy as a benchmark for other carcinomas for which lymphadenectomy has been established as the standard surgical procedure. Furthermore, we adopted simple zoning of each lymph node station around the liver with the lymphatic drainage route and practical use of lymphadenectomy. According to these classifications of lymph node, the implications of Level 2 include more accurate staging than Level 1,^18^ while those of Level 3 include a left pathway or cardinal route as a higher likelihood of lymphatic basin, particularly for left lateral ICCs.^30^, ^31^, ^32^

In our results, the incidence of LNM heavily depended on tumor location: hilar lesions showed the highest LNM potential, followed by left peripheral and right peripheral lesions. There was no doubt that Level 1 had the most frequent nodes as a primary lymph node station, and Level 2 could represent possible metastatic nodes from the left peripheral and hilar regions. On the other hand, the right peripheral region seldom showed lymphatic spread to Level 3. In our cohort, very few Level 2‐LNM or Level 3‐LNM were seen without Level 1‐LNM. In other words, Level 2‐ or Level 3‐LNM is usually accompanied by Level 1‐LNM. Therefore, Level 3 as a left pathway or cardinal route should be considered as an independent lymphatic route, particularly for left lateral ICCs, but in terms of therapeutic efficacy and staging significance, should be regarded as second only to Level 1 and Level 2.

Concerning therapeutic value, the 5‐year TI of lymphadenectomy was 7.2 for Level 1, 5.5 for Level 2, and 1.9 for Level 3. According to tumor localization, the hilar lesion showed 5‐year TI > 5.0 in Level 1 and Level 2. On the other hand, bilateral peripheral lesions were only seen in Level 1. Based on these results, lymphadenectomy for Levels 1 and 2 could exert a positive impact on prognosis for hilar lesions. As well as this, lymphadenectomy for Level 1 could have a positive impact on peripheral lesions. To justify the indications for lymphadenectomy, future work should examine where best to set the cut‐off for TI. Although the efficacy would be limited, lymphadenectomy for Level 3 in the left peripheral region is worth considering.

Adjuvant therapy has recently been considered more closely for further achieving improvements of the surgical prognosis for ICC. While the clinical benefits of adjuvant treatment for ICC remain unclear, the potential survival benefits of adjuvant chemotherapy could be associated with tumor subgroups, such as the presence of LNM.^33^ From this perspective, accurate staging through lymphadenectomy is indispensable. Regarding the precise stage for estimating the degree of cancer spread, Level 1‐ to 3‐LNM could reflect the degree of cancer spread. Multisite recurrence, including to the remnant liver, lymph nodes, and other organs, could relate to lymphatic spread. Expanding the range of lymphadenectomy could thus lead to more accurate staging.

Nonetheless, it must be noted that lymphadenectomy is also associated with a specific risk of postoperative morbidity. Postoperative morbidity would possibly delay the introduction of adjuvant chemotherapy. Extending lymphadenectomy to Level 3 could increase the occurrence of CD Grade II complications, among which gastric stasis is prominent. These patients required longer hospitalization and delayed introduction of adjuvant chemotherapy. Preemptive pyloroplasty may help prevent this situation.^34^ However, the indications for lymphadenectomy for Level 3 disease should be considered based on the balance between therapeutic effects and the significance of staging and its disadvantages. Consequently, determination of the extent of, and indications for, lymph node dissection is essential, taking into account the therapeutic and diagnostic implications according to the location of the ICC.

Some limitations to this study should be kept in mind. This analysis focused on TI to confirm the clinical implications of lymphadenectomy for ICC. However, nodal status may migrate in cases where the extent of routine lymphadenectomy would be different in a retrospective, multicenter collection of cases. Therefore, even though more than 80% of the patients in this study had undergone Level 2 or higher lymphadenectomy, it is important to note that the patient group is comprised of a heterogeneous population with an extent of dissection. Furthermore, the actual TIs were also indicated in Level 2 and 3 lymph nodes. It should be noted, however, that TI could be calculated based on the survival rate of very rare positive metastases, as seen in the peripheral‐type of pancreatic retroperitoneal lymph nodes.

These results may be difficult to accept because of previous reports referring to dismal results from lymphadenectomy. However, our results suggest that a certain number of patients will gain therapeutic value from lymphadenectomy significant enough to determine the exact extent of ICC. Moreover, the classification of lymph node level could contribute to determination of the extent of lymphadenectomy. We report the therapeutic value and clinical implications of lymphadenectomy for ICC differently from previous reports. Further, tumor location could offer a significant determinant for the priority of lymphadenectomy.

## 5 CONCLUSIONS

Lymphadenectomy achieved by liver resection with curative intent should be a minimal requirement for the multimodal treatment of ICC. Furthermore, the implications and extent of lymphadenectomy for ICC should be determined according to the tumor location and preoperative risk of LNM. In peripheral type ICC, the prognostic benefit of lymphadenectomy would be limited and dissection beyond level 1 should be eliminated, while in hilar‐type, lymphadenectomy up to level 2 would be necessary.

## AUTHOR CONTRIBUTIONS

Yuzo Umeda: Study design, data analysis, and writing the article. Kosei Takagi, Tomokazu Fuji, and Tatsuo Matsuda: Data analysis. Toru Kojima, Daisuke Satoh, Masayoshi Hioki, Yoshikatsu Endo, Masaru Inagaki, Masahiro Oishi, Takahito Yagi, and Toshiyoshi Fujiwara: Data collection.

## FUNDING INFORMATION

Financial support was received from the Japan Society for the Promotion of Science (grant number 22 K08775 to Yuzo UMEDA).

## CONFLICT OF INTEREST

The authors declare no conflicts of interest for this article.

## ETHICS STATEMENT

Approval of the research protocol: This study conformed to the Declaration of Helsinki on Human Research Ethics standards and was approved by the Okayama University Hospital Institutional Ethics Board (approval no. 2208–016).

Informed Consent: The need for written, informed consent was waived by the Okayama University Hospital Institutional Ethics Board because of the retrospective design of this study.

Registry and the Registration No. of the study/trial: N/A.

Animal Studies: N/A.

## Supporting information


Figure S1

Figure S2

Figure S3

Table S1

## Data Availability

The datasets generated and/or analyzed during the present study are not publicly available due to the data privacy policy at our facility, but are available from the corresponding author on reasonable request.
