# Supplementary figures and images for: Clinical implications and optimal extent of lymphadenectomy for intrahepatic cholangiocarcinoma: A multicenter analysis of the therapeutic index
Source: Ann Gastroenterol Surg. 2022 Nov 27;7(3):512–22. doi: 10.1002/ags3.12642 (PMC10154828; doi:10.1002/ags3.12642)

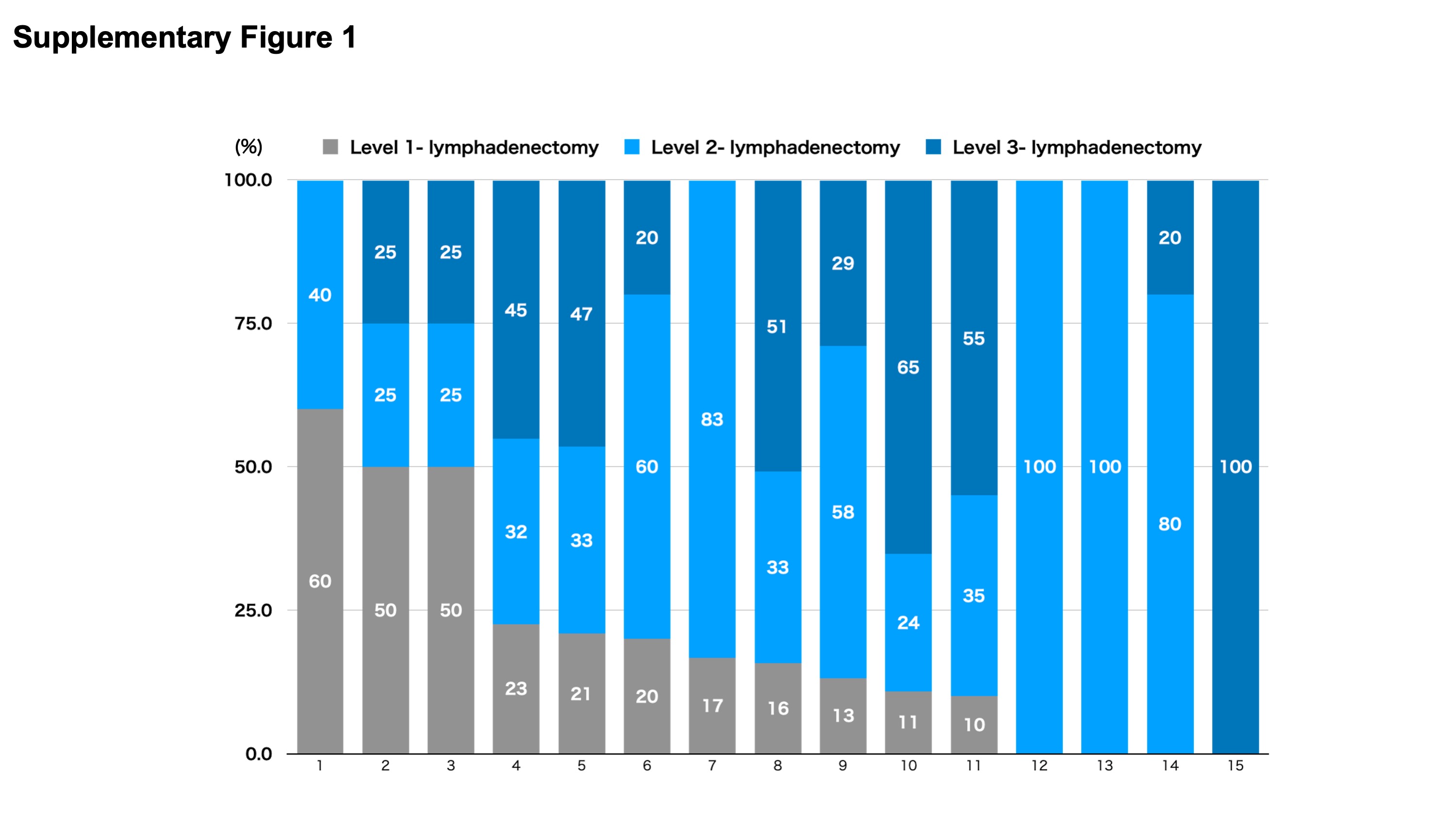

Supplement: Supplementary file 1 — Figure S1 [file AGS3-7-512-s004.jpeg]

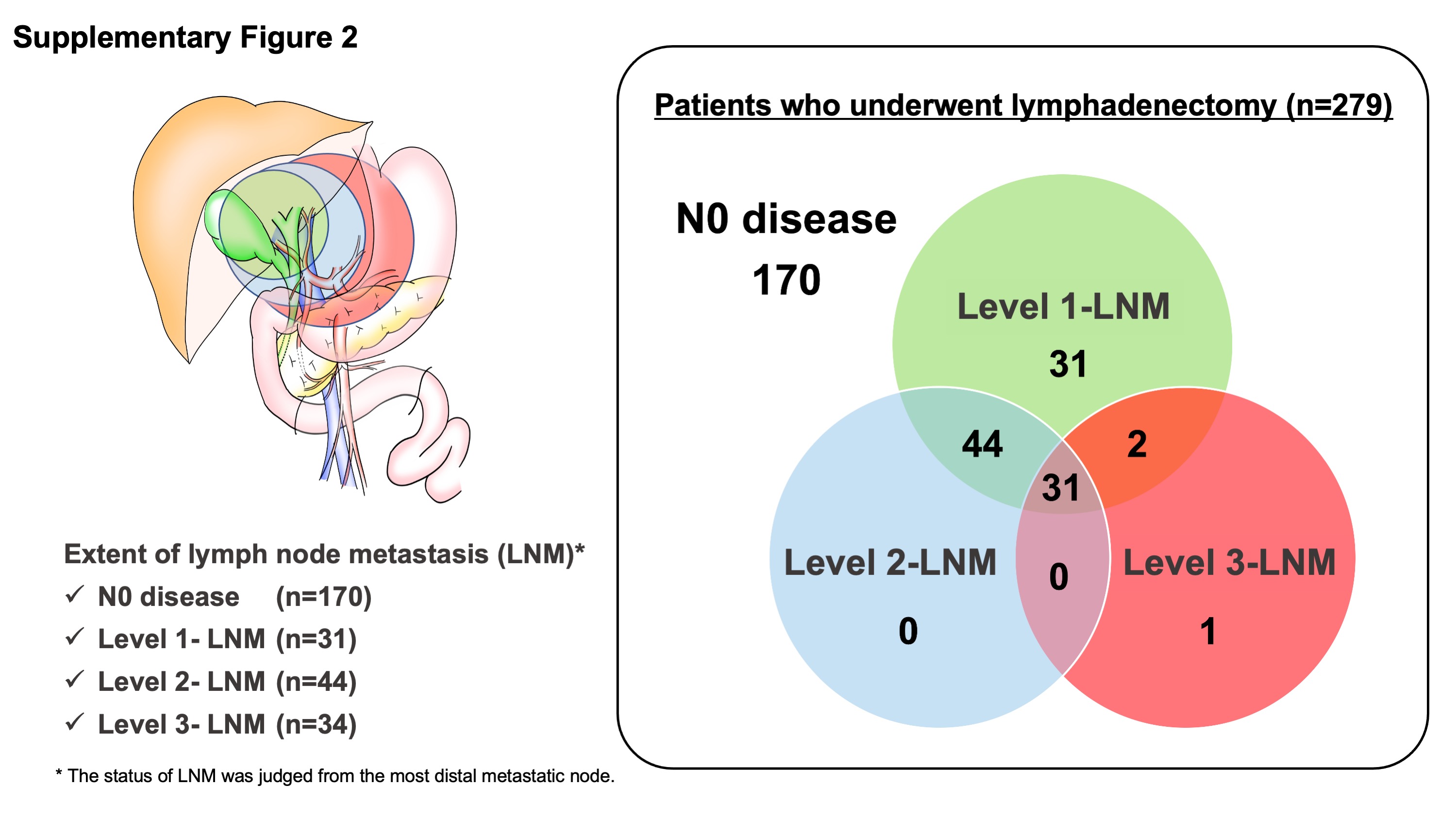

Supplement: Supplementary file 2 — Figure S2 [file AGS3-7-512-s003.jpeg]

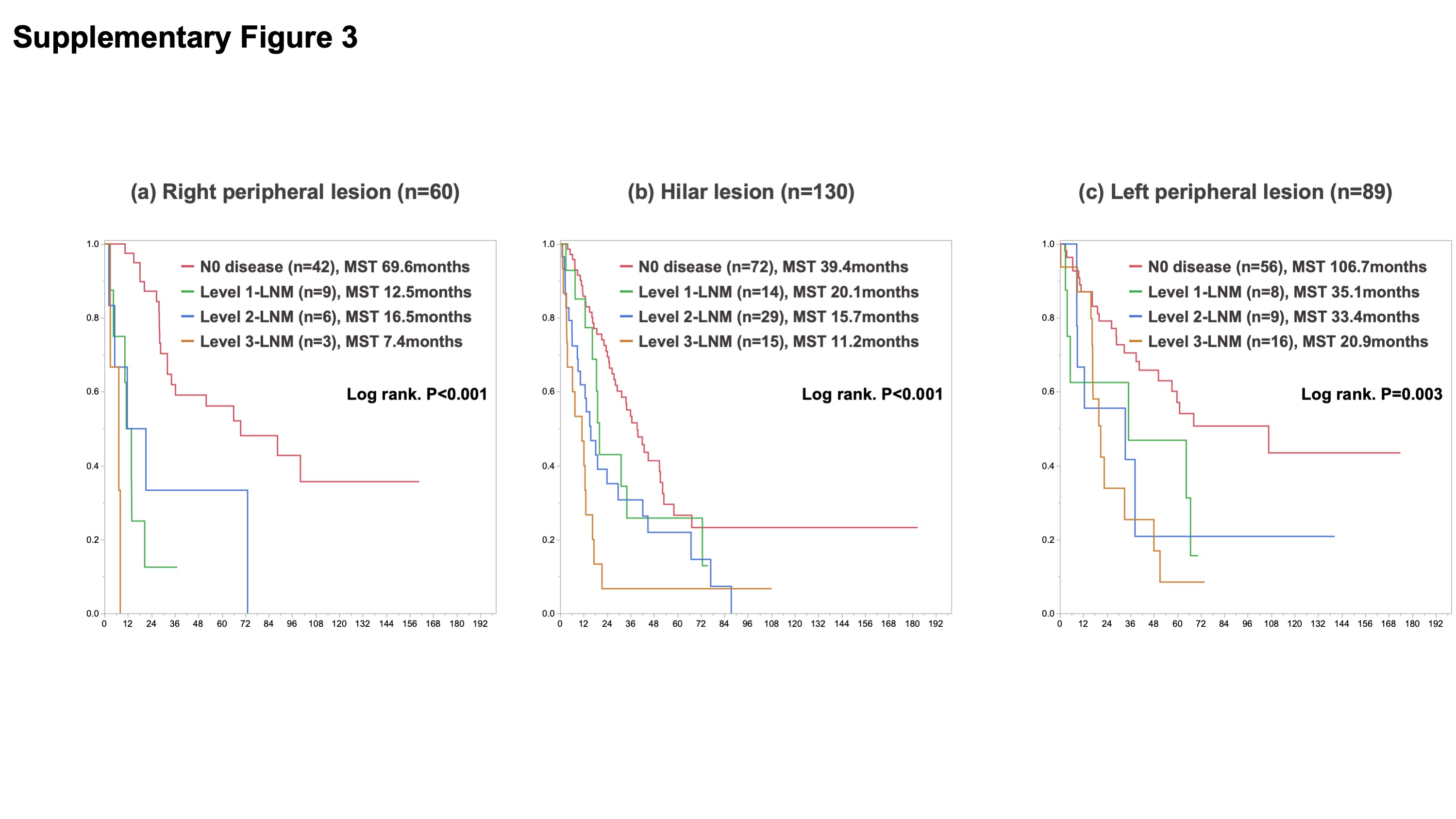

Supplement: Supplementary file 3 — Figure S3 [file AGS3-7-512-s002.jpeg]
