# Supplementary material for: Clinical implications and optimal extent of lymphadenectomy for intrahepatic cholangiocarcinoma: A multicenter analysis of the therapeutic index
Source: Ann Gastroenterol Surg. 2022 Nov 27;7(3):512–22. doi: 10.1002/ags3.12642 (PMC10154828; doi:10.1002/ags3.12642)
Supplement: Supplementary file 4 — Table S1 [file AGS3-7-512-s001.docx]

| **Supplementary table 1.** Logistic regression analysis to examine risk factors for lymph node metastasis (n=279) | | | | | | | |
| --- | --- | --- | --- | --- | --- | --- | --- |
| **Variables** | | **Univariate analysis** | | | **Multivariate analysis** | | |
|  |  | **Odds ratio** | **95% CI** | **P-value** | **Odds ratio** | **95% CI** | **P-value** |
| **Background factor** | |  |  |  |  |  |  |
|  | Gender |  |  |  |  |  |  |
|  | male | 1.00 (reference) |  |  |  |  |  |
|  | female | 1.36 | 0.84-2.22 | 0.205 |  |  |  |
|  | Age |  |  |  |  |  |  |
|  | <60 years | 1.00 (reference) |  |  |  |  |  |
|  | ≥60 years | 1.64 | 0.81-3.30 | 0.162 |  |  |  |
| **Tumor factor** | |  |  |  |  |  |  |
|  | Tumor morphology |  |  |  |  |  |  |
|  | MF | 1.00 (reference) |  |  | 1.00 (reference) |  |  |
|  | PI | 2.31 | 1.34-3.99 | 0.002 | 2.90 | 1.46-5.76 | 0.002 |
|  | Tumor size |  |  |  |  |  |  |
|  | < 5cm | 1.00 (reference) |  |  | 1.00 (reference) |  |  |
|  | ≥ 5cm | 1.37 | 1.00-1.89 | 0.046 | 1.44 | 0.79-2.63 | 0.227 |
|  | Tumor location |  |  |  |  |  |  |
|  | Peripheral | 1.00 (reference) |  |  | 1.00 (reference) |  |  |
|  | Hilar | 1.54 | 0.95-2.51 | 0.076 | 1.26 | 0.70-2.25 | 0.430 |
|  | Multinodular |  |  |  |  |  |  |
|  | no | 1.00 (reference) |  |  |  |  |  |
|  | yes | 1.28 | 0.73-2.26 | 0.383 |  |  |  |
|  | CA19-9 |  |  |  |  |  |  |
|  | <118U/mL | 1.00 (reference) |  |  | 1.00 (reference) |  |  |
|  | ≥118U/mL | 6.02 | 3.42-10.56 | <0.001 | 6.77 | 3.63-12.6 | <0.001 |
|  | Serosa invasion |  |  |  |  |  |  |
|  | no | 1.00 (reference) |  |  | 1.00 (reference) |  |  |
|  | yes | 1.93 | 1.18-3.14 | 0.008 | 1.81 | 1.02-3.23 | 0.043 |
|  | Vascular invasion |  |  |  |  |  |  |
|  | no | 1.00 (reference) |  |  |  |  |  |
|  | yes | 1.41 | 0.87-2.31 | 0.161 |  |  |  |
|  | Differentiation |  |  |  |  |  |  |
|  | well | 1.00 (reference) | 1.05-1.64 |  | 1.00 (reference) | 1.05-1.64 |  |
|  | moderate/poor | 3.11 | 1.62-5.99 | 0.008 | 4.51 | 2.10-9.86 | 0.001 |
|  | unclassified | 1.85 | 0.59-5.83 | 0.288 | 3.22 | 0.81-12.7 | 0.094 |
| **Abbreviations:** BMI. body mass index; CA19-9. carbohydrate antigen 19-9; CI. confidence interval; MF mass-forming; PI. periductal infiltrating | | | | | | |  |
